# Steroidogenesis Upregulation through Mitochondria-Associated Endoplasmic Reticulum Membranes and Mitochondrial Dynamics in Rat Testes: The Role of D-Aspartate

**DOI:** 10.3390/cells13060523

**Published:** 2024-03-16

**Authors:** Debora Latino, Massimo Venditti, Sara Falvo, Giulia Grillo, Alessandra Santillo, Imed Messaoudi, Mariem Ben Rhouma, Sergio Minucci, Gabriella Chieffi Baccari, Maria Maddalena Di Fiore

**Affiliations:** 1 Department of Environmental, Biological and Pharmaceutical Sciences and Technologies, University of Campania ‘Luigi Vanvitelli’, 81100 Caserta, Italy; debora.latino@unicampania.it (D.L.); sara.falvo@unicampania.it (S.F.); giulia.grillo@unicampania.it (G.G.); alessandra.santillo@unicampania.it (A.S.); gabriella.chieffi@unicampania.it (G.C.B.); 2 Department of Experimental Medicine, Section Human Physiology and Integrated Biological Functions, University of Campania ‘Luigi Vanvitelli’, 80138 Napoli, Italy; massimo.venditti@unicampania.it (M.V.); sergio.minucci@unicampania.it (S.M.); 3 LR11ES41: Génetique, Biodiversité et Valorisation des Bioressources, Institut Supérieur de Biotechnologie, Université de Monastir, Monastir 5000, Tunisia; imed_messaoudi@yahoo.fr (I.M.); benrhoumamariem95@gmail.com (M.B.R.)

**Keywords:** endoplasmic reticulum, mitochondria, steroidogenesis, lipid traffic, calcium signaling, ER stress, mitochondrial dynamics, fusion, fission, biogenesis, mass

## Abstract

Mitochondria-Associated Endoplasmic Reticulum Membranes (MAMs) mediate the communication between the Endoplasmic Reticulum (ER) and the mitochondria, playing a fundamental role in steroidogenesis. This study aimed to understand how D-aspartate (D-Asp), a well-known stimulator of testosterone biosynthesis and spermatogenesis, affects the mechanism of steroidogenesis in rat testes. Our results suggested that D-Asp exerts this function through MAMs, affecting lipid trafficking, calcium signaling, ER stress, and mitochondrial dynamics. After 15 days of oral administration of D-Asp to rats, there was an increase in both antioxidant enzymes (SOD and Catalase) and in the protein expression levels of ATAD3A, FACL4, and SOAT1, which are markers of lipid transfer, as well as VDAC and GRP75, which are markers of calcium signaling. Additionally, there was a decrease in protein expression levels of GRP78, a marker of aging that counteracts ER stress. The effects of D-Asp on mitochondrial dynamics strongly suggested its active role as well. It induced the expression levels of proteins involved in fusion (MFN1, MFN2, and OPA1) and in biogenesis (NRF1 and TFAM), as well as in mitochondrial mass (TOMM20), and decreased the expression level of DRP1, a crucial mitochondrial fission marker. These findings suggested D-Asp involvement in the functional improvement of mitochondria during steroidogenesis. Immunofluorescent signals of ATAD3A, MFN1/2, TFAM, and TOMM20 confirmed their localization in Leydig cells showing an intensity upgrade in D-Asp-treated rat testes. Taken together, our results demonstrate the involvement of D-Asp in the steroidogenesis of rat testes, acting at multiple stages of both MAMs and mitochondrial dynamics, opening new opportunities for future investigation in other steroidogenic tissues.

## 1. Introduction

The current knowledge on the functions of Mitochondria-Associated Endoplasmic Reticulum Membranes (MAMs) in human health found a relevant association between dysregulation of MAMs and different pathological conditions, such as diabetes, cancer and neurodegenerative diseases [1]. This is the first study to explore the role of MAMs in steroidogenesis under experimental condition. The multi-enzymatic process of steroidogenesis converts cholesterol into biologically-active steroid hormones via communication between the Endoplasmic Reticulum (ER) and mitochondria occurring through mitochondria-associated membranes. MAMs play essential roles in many cellular functions, including lipid metabolism and transfer, calcium signaling, and mitochondrial morphology and dynamics, which are critical for steroidogenesis [2]. At the first step of steroidogenesis, cholesterol is transported from the ER to the mitochondria, where it is converted into steroids. Some detailed reviews describe the role of MAMs in lipid metabolism and transport between ER and mitochondria [3,4], suggesting that close contact sites between the ER and mitochondria act as bridges for lipid translocation. Among the array of proteins involved, there are ATPase family AAA Domain-containing protein 3 (ATAD3A), which facilitates the interaction between ER and mitochondria, regulating cholesterol transport and ER stress; fatty acid-CoA ligase 4 (FACL4), which mediates the synthesis of cellular lipids and cholesterol metabolites; Sterol O-Acyltransferase 1 (ACAT1/SOAT1), which converts free cholesterol to cholesterol ester for its storage. It is known that steroidogenesis also depends on mitochondrial calcium levels; some proteins, such as voltage-dependent anion channel (VDAC) and Glucose-Regulated Protein 75 (GRP75), are localized on MAMs and regulate Ca^2+^ release from ER for its efficient mitochondrial uptake [5]. In addition, calcium contributes either to intra-mitochondrial cholesterol transfer or to the steroidogenic acute regulatory (StAR) protein function in cholesterol transport from the outer mitochondrial membrane (OMM) to the inner mitochondrial membrane (IMM) [6]. The Glucose-Regulated Protein 78 (GRP78) is a heat shock protein that acts as an important chaperone, facilitates protein folding, and prevents the accumulation of protein aggregates in the ER [7]. It is the master regulator for ER stress; in fact, GRP78 transcription is induced in conjunction with ER stress, and its increase is linked to this state. Several findings suggest that structural changes in mitochondria are involved in steroidogenesis, indicating a link between steroidogenesis and mitochondrial structure. Mitochondria dynamic changes regarding fission, fusion, and biogenesis are all correlated with mitochondrial mass. This plasticity provides mitochondria with the flexibility needed to respond to cellular stresses. In this regard, several proteins are involved in such dynamic activities: Dynamin-associated protein 1 (DRP1) regulates mitochondrial fission, while Mitofusin 1 and 2 (MFN1 and MFN2), dynamin-like GTPases, and the protein optic atrophy 1 (OPA1), are involved in mitochondrial fusion [8]. In addition, MFN2 is also implicated in modulating interactions between mitochondria and the ER. All the above-mentioned proteins play important regulatory roles during steroidogenesis [9]. To ensure optimal function, quality control is regulated through mitochondrial turnover, which includes mitochondrial biogenesis, a process involving the growth and division of existing mitochondria and the genesis of new ones. Two factors play significant roles in mitochondrial biogenesis: the Nuclear Respiratory Factor 1 (NRF1) and the Mitochondrial Transcription Factor A (TFAM) [10,11]. NRF1 upregulates the expression of nuclear genes encoding TFAM and other mitochondrial proteins. TFAM is then transported into mitochondria where it binds to mitochondrial DNA (mtDNA) and activates the transcription and replication of the mtDNA essential for the generation of new mitochondria. Taken together, all these dynamic processes regulate the mitochondrial mass, which refers to the total quantity of this organelle within a cell. The protein Translocase of the Outer Mitochondrial Membrane 20 (TOMM20) is an essential component of the TOM complex, responsible for importing proteins into the mitochondria [12]. Therefore, the levels of TOMM20 in the outer mitochondrial membrane can influence mitochondrial mass and function, which is crucial for maintaining their health and proper functioning.

Testicular steroidogenesis has been widely demonstrated to be influenced by various factors. One molecule that has gained significant attention in the last two decades is D-aspartate (D-Asp), an amino acid that is naturally present in the testes of vertebrates [13,14,15] performing reproductive functions. Several studies conducted both in vivo and in vitro have shown that D-Asp induces the synthesis of testosterone (T) in rat Leydig cells by increasing the levels of cAMP, activating the expression of StAR protein and of the steroidogenic enzymes, such as 3β-hydroxysteroid dehydrogenase (3β-HSD) and 17β-hydroxysteroid dehydrogenase (17β-HSD) [13]. Despite a growing body of literature on D-Asp and its role in steroidogenesis and spermatogenesis, little is known about the molecular mechanisms underlying the action of the amino acid in rat testes.

This study aims to understand the role of D-aspartate in steroidogenesis by investigating its effects on the overall process, which is closely linked to the functionality of the mitochondria and their efficient communication with the endoplasmic reticulum via the MAMs. In this study, we have shown for the first time that D-Asp can improve the steroidogenic process in rat testes by acting directly on the mitochondrial compartment and on the MAMs.

## 2. Materials and Methods

### 2.1. Experimental Design

In this study, male Wistar rats (*n* = 10) aged 60 days were housed in individual cages under controlled conditions of 12:12 L/D cycle, a temperature of 22 ± 1 °C, and humidity of 55 ± 15%. They were fed with a standard rodent diet and with drinking water ad libitum. The animals were divided into two groups with five animals in each group. Control group (C) received 2 mL of distilled water/day/rat by gastric gavage for 15 days; D-Asp group (D-Asp) received 0.1 mM D-Asp/day/g body weight by gastric gavage for 15 days (D-Asp; 219096; Sigma-Aldrich, Milan, Italy). The rats were weighed every 5 days. D-Asp administration dosage and route was chosen based on numerous experiments [14] showing that the administration of 0.1 mM D-Asp/day/g body weight for 15 days determines the required amino acid uptake necessary to activate steroidogenesis in the testes.

### 2.2. Sample Collection

After experimental treatment, rats were euthanized 24 h after gavage. From each animal, blood was collected by cardiac puncture, placed in sterile tubes, and centrifuged at 3000 rpm for 10 min at 4 °C. The obtained serum was stored at −80 °C for hormone determination. The testes were removed and weighed. One testis was fixed in 10% formalin for Immunofluorescence (IF) investigations, and the other was stored at −80 °C for molecular analyses.

### 2.3. Determination of Serum T Levels

Serum testosterone levels were assayed by ELISA method using a commercial kit (DK0002, Diametra, Milan, Italy).

### 2.4. Oxidative Stress

The enzymatic activities of Superoxide dismutase (SOD) and Catalases (CAT) were measured according to the methods of [16,17] and expressed as U/mg protein. Testis lysates (see “Protein extraction”) were used to determine thiobarbituric acid reactive substance (TBARS) levels, following a previous publication [18]. The results were expressed as TBARS (μM/μg extracted protein).

### 2.5. Protein Extraction and Western Blot (WB) Analysis

Each testis was processed according to [18,19] protocols for protein extraction and for WB analysis. Table 1 reports the details of all primary and secondary antibodies used. ImageJ software (version 1.53 t; National Institutes of Health, Bethesda, MD, USA) was used for protein quantification.

### 2.6. IF Analysis

For ATAD3A, MFN1, MFN2, TFAM, SYCP3, StAR, and TOMM20 localization analysis, 5 µm testis sections were dewaxed, rehydrated, and incubated overnight with primary antibodies anti-ATAD3A, anti-TFAM, anti-MFN1, anti-MFN2, anti-SYCP3, anti-StAR, anti-TOMM20, and anti-β-Actin, at 4 °C. After three washes in PBS, slides were incubated with their respective secondary antibodies and with peanut agglutin (PNA) lectin for 1 h at room temperature. Nuclei were stained with Vectashield + DAPI, and slides were observed and captured with an optical microscope (Leica DM 5000 B (Wetzlar, Germany) + CTR 5000) with a UV lamp and saved with IM 1000 software. Densitometric analysis of ATAD3A, TFAM, MFN1, MFN2, and TOMM20 immunofluorescence signal was performed with Fiji plugin (version 3.9.0/1.53 t) of ImageJ Software counting 20 seminiferous tubules per animal for a total of 100 tubules per group.

### 2.7. Statistical Analysis

The analysis of variance followed by the Student–Newman–Keuls test assessed the significant changes between the experimental groups. Values less than *p* < 0.05 were considered statistically significant. All data were shown as the mean ± S.D. (standard deviation).

## 3. Results

### 3.1. Determination of Serum Testosterone Levels

Serum testosterone levels in D-Asp-treated rats (5.2 ± 0.2 ng/mL) were significantly higher (*p* < 0.01) than those in the controls (3.6 ± 0.4 ng/mL).

### 3.2. Effects of D-Asp on Oxidative Stress

**Figure 1:**
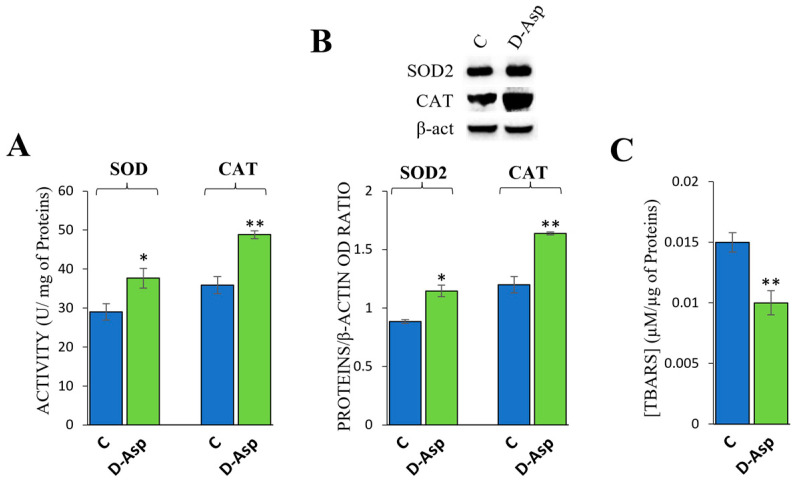
Effects of D-Asp treatment on oxidative stress parameters. Evaluation of testicular enzymatic activities (**A**) and protein expression levels of SOD2 (25 kDa) and CAT (60 kDa) (**B**) in the testes of rats treated with D-Asp and compared to controls. The expression levels of SOD2 and CAT were quantified using ImageJ and normalized to β-actin (42 kDa). Evaluation of testicular TBARS levels of rats exposed to D-Asp compared to controls (**C**). All values are expressed as the mean ± SD of 5 animals in each group. D-Asp vs. Control (C) * *p* < 0.05; ** *p* < 0.01.

The investigation of oxidative stress was essential to determine whether D-Asp could have harmful effects on testicular cells. The direct measurement of the activity of the most important antioxidant enzymes is a relevant method to determine the conditions of oxidative stress. Interestingly, D-Asp significantly increased superoxide dismutase (*p* < 0.05) and Catalase (*p* < 0.01) activities compared to controls (Figure 1A). Additionally, D-Asp increased the protein expression levels of the antioxidant enzymes SOD2 (*p* < 0.05) and CAT (*p* < 0.01), compared to the control (Figure 1B). Oxidative stress was also analyzed by measuring testicular levels of TBARS as an index of lipid peroxidation. D-Asp treatment caused a significant decrease in TBARS levels compared to the controls (*p* < 0.01, Figure 1C).

### 3.3. MAMs and Mitochondrial Compartment

To investigate the effects of the cellular mechanisms activated by D-Asp on the steroidogenesis of rat testes, we examined the expression of key proteins involved in both MAMs and mitochondrial compartments. Specifically, we focused on the effect of D-Asp on the interactions between the ER and mitochondria, which are linked by several cellular processes, including lipid transfer and calcium signaling.

#### 3.3.1. Effects of D-Asp on Lipid Transfer

**Figure 2:**
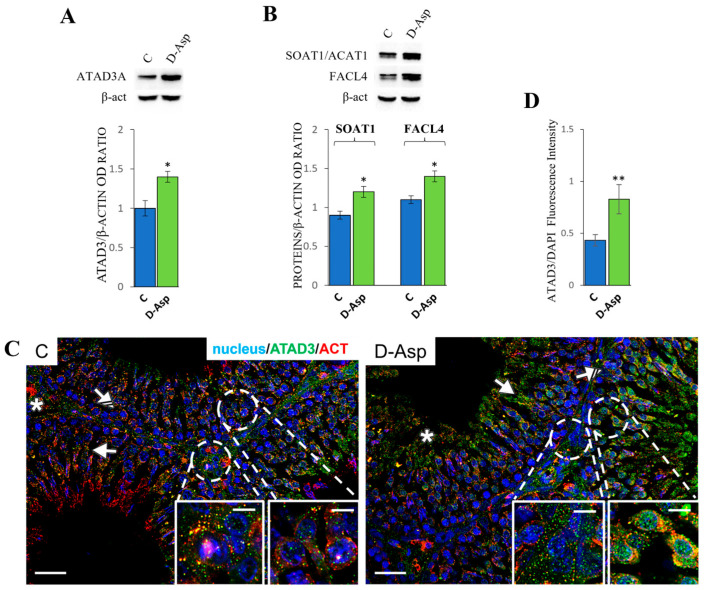
Lipid transfer analysis of control and D-Asp-treated rats. (**A**) Western blot analysis of ATAD3A (70 kDa), (**B**) SOAT1 (47 kDa), and FACL4 (79 kDa) protein levels in the testes of animals treated with D-Asp for 15 days compared to controls. The expression levels of ATAD3A, SOAT1, and FACL4 were quantified using ImageJ and normalized to β-actin (42 kDa). All values are expressed as the mean ± SD of 5 animals in each group. D-Asp vs. Control (C): * *p* < 0.05. (**C**) Immunofluorescence analysis of ATAD3A (green), β-actin (red), and nucleus (blue) from control and D-Asp-treated rats. Striped arrows: spermatocytes; solid arrows: spermatids; asterisks: Leydig cells. Scale bars represent 20 μm and 10 μm in the insets. (**D**) Histogram showing quantification of ATAD3A fluorescence signal intensity. All values are expressed as the mean ± SD of 5 animals in each group. D-Asp vs. Control (C): ** *p* < 0.01.

The lipid transfer of cholesterol from the ER to the mitochondria represents a fundamental step in steroidogenesis. As reported above, the proteins ATAD3A, SOAT1, and FACL4 are involved in this process. The expression levels of these proteins were evaluated in the rat testes of the experimental and control groups (Figure 2A,B). The results of the WB analyses showed that D-Asp treatment for 15 days increased the protein levels of ATAD3A (*p* < 0.05) (Figure 2A), SOAT1 (*p* < 0.05), and FACL4 (*p* < 0.05) compared to the respective control groups (Figure 2B). The analyses indicated that D-Asp treatment promoted cholesterol transfer from the ER to the mitochondria. To confirm the above data and to analyze the localization of ATAD3A, we performed immunofluorescence staining in the same groups (Figure 2C). The ATAD3A signal was diffuse in all cells of the seminiferous epithelium, including the interstitial Leydig cells (asterisks; left inset), the perinuclear space of spermatocytes (striped arrows; right inset), and the cytoplasm of elongating spermatids (arrows) in both the control and D-Asp-treated groups. However, the signal was stronger in the D-Asp group than in the controls (*p* < 0.01; Figure 2D), as determined by the fluorescence intensity analysis.

#### 3.3.2. Effects of D-Asp on Ca^2+^ Signaling and ER Stress

**Figure 3:**
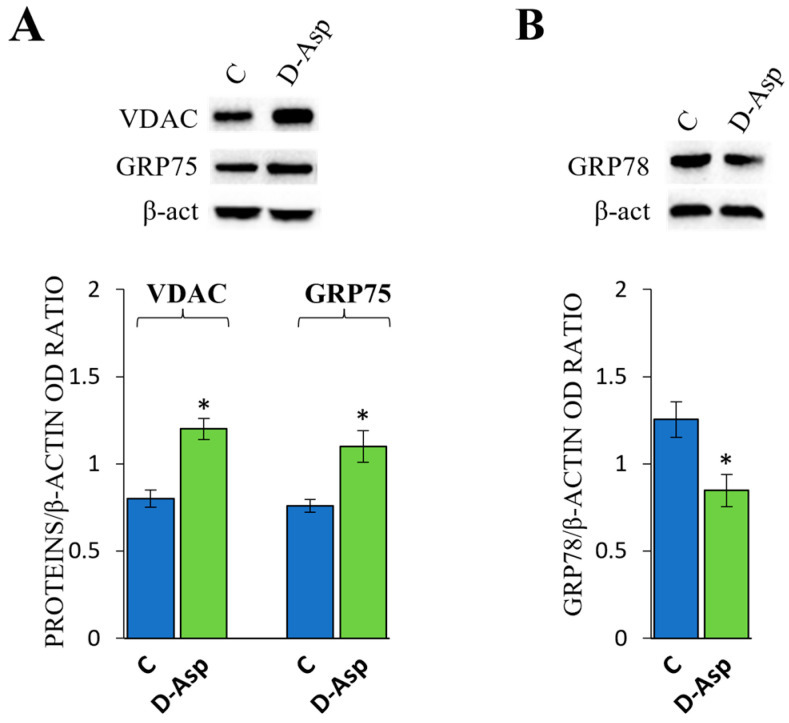
Analysis of calcium signaling (**A**) and ER stress (**B**) in control and D-Asp-treated rats. (**A**) Western blot analysis of VDAC (32 kDa) and GRP75 (75 kDa) protein levels in the testes of animals treated with D-Asp for 15 days compared to controls. (**B**) Western blot analysis of GRP78 (78 kDa) protein levels in rat testes of D-Asp group compared to controls. The expression levels of VDAC, GRP75, and GRP78 were quantified using ImageJ and normalized to β-actin (42 kDa). All values are expressed as the mean ± SD of 5 animals in each group. D-Asp vs. Control (C): * *p* < 0.05.

The importance of the Ca^2+^ signaling pathway has been established in several steroid hormone syntheses and male fertility; particularly, the Ca^2+^ molecular pathway is considered essential for steroidogenesis in Leydig cells [20]. To assess the potential role of D-Asp in regulating Ca^2+^ signaling, we examined the protein levels of VDAC and GRP75 (Figure 3A). Additionally, we analyzed the expression levels of GRP78, a major ER stress marker, to investigate whether any changes in calcium transfer from the ER to mitochondria after D-Asp treatment could affect the oxidative state of the ER (Figure 3B). The treatment with D-Asp resulted in a significant increase in the protein levels of VDAC and GRP75 (*p* < 0.05; Figure 3A). Conversely, a significant decrease in the expression of GRP78 (*p* < 0.05) was observed compared to the controls (Figure 3B).

### 3.4. Mitochondrial Dynamics

The mitochondria represent the key organelles within which the steroidogenetic process begins. Therefore, the correct arrangement of these organelles is of crucial importance for right hormone synthesis. Mitochondrial dynamics including fission, fusion, and biogenesis are all correlated with mitochondrial mass.

#### 3.4.1. Effects of D-Asp on Fusion and Fission

**Figure 4:**
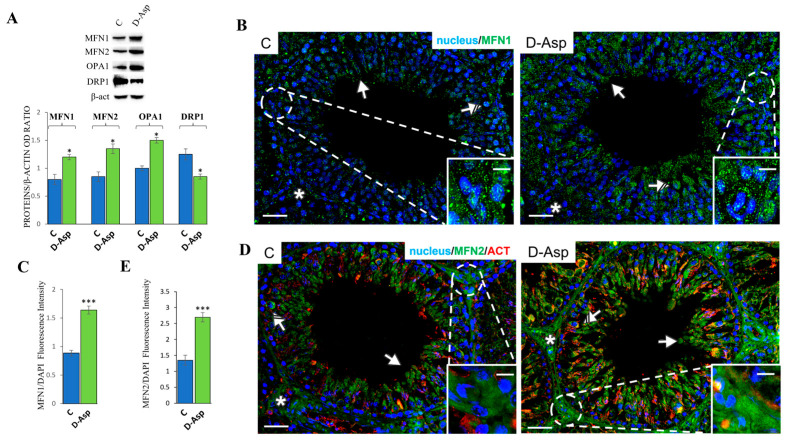
Analysis of mitochondrial fusion and fission of control and D-Asp-treated rats. (**A**) Western blot analysis of MFN1 (84 kDa), MFN2 (86 kDa), OPA1 (93–111 kDa), and DRP1 (78–82 kDa) protein expression levels in the testes of rats treated with D-Asp for 15 days, compared to controls. The expression levels of MFN1, MFN2, OPA1, and DRP1 were quantified using ImageJ and normalized to β-actin (42 kDa). (**B**) Immunofluorescence analysis of MFN1 (green) and nucleus (blue) of control and D-Asp-treated rats. (**D**) Immunofluorescence analysis of MFN2 (green), β-actin (red), and nucleus (blue) of control and D-Asp-treated rats. Striped Arrows: spermatocytes; solid arrows: spermatids; asterisks: Leydig cells. Scale bars represent 20 μm and 10 μm in the insets. Histogram showing the quantification of MFN1 (**C**) and MFN2 (**E**) fluorescence signal intensity. All values are expressed as mean ± SD of 5 animals in each group. D-Asp vs. Control (C): * *p* < 0.05; *** *p* < 0.001.

The effects of D-Asp treatment on the mitochondrial compartment were evaluated by examining its involvement in mitochondrial dynamics, specifically fusion and fission (Figure 4).

The expression levels of MFN1, MFN2, and optic atrophy 1 (OPA1) were examined to assess the effects of D-Asp administration on mitochondrial fusion. WB analyses revealed that treatment with D-Asp resulted in a significant increase in MFN1, MFN2, and OPA1 expression levels compared to the respective control groups (*p* < 0.05; Figure 4A). To investigate the effect of D-Asp on mitochondrial fission, we estimated the expression levels of DRP1. The results showed that DRP1 expression levels were lower in the D-Asp group than in the control group (*p* < 0.05; Figure 4A), suggesting that D-Asp treatment may shift the mitochondrial dynamics in the direction of fusion rather than fission. To determine the localization of MFN1 and MFN2, immunofluorescence staining was performed in both the control and D-Asp-treated testes (Figure 4B,D). As shown in Figure 4B, in the control testes, MFN1 was localized diffusely in the cytoplasm of germ cells, particularly in spermatocytes (striped arrows; inset) and round spermatids (solid arrows). Positive staining was also observed in the interstitial Leydig cells (asterisks). In the testis sections of D-Asp-treated rats, a significant increase in staining was observed in most germ cells, and a strong signal was detected in the cytoplasm of Leydig cells. The fluorescence intensity analysis confirmed a significant increase in MFN1 levels (*p* < 0.001) (Figure 4C) in the D-Asp group compared to the control group. MFN2 was localized in all cells of the seminiferous epithelium, particularly in the interstitial Leydig cells (asterisks; inset), spermatocytes (striped arrows; right inset), and elongating spermatid cytoplasm (arrows) in both control and D-Asp-treated groups (Figure 4D). However, the signal appeared stronger in the D-Asp group than in the controls, particularly in the cytoplasm of Leydig cells (*p* < 0.001; Figure 4E), as determined by the fluorescence intensity analysis.

#### 3.4.2. Effects of D-Asp on Mitochondrial Biogenesis

**Figure 5:**
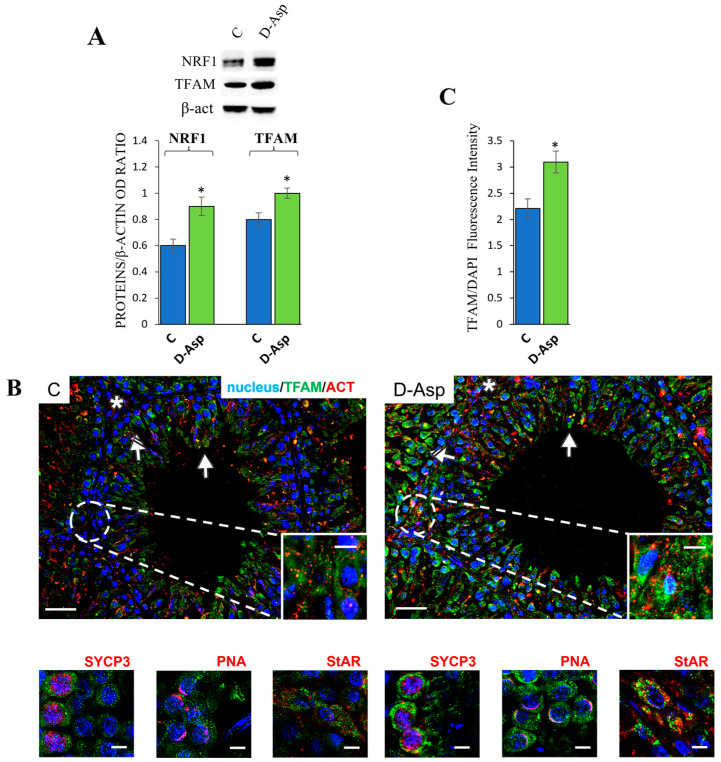
Mitochondrial biogenesis analysis of control and D-Asp-treated rats. (**A**) Western blot analysis of NRF1 (56 kDa) and TFAM (28 kDa) protein expression levels in the testes of rats treated with D-Asp for 15 days compared to controls. The expression levels of NRF1 and TFAM were quantified using ImageJ and normalized to β-actin (42 kDa). (**B**) Immunofluorescence analysis of TFAM (green), β-actin (red), and nucleus (blue) from control and D-Asp-treated rats. Striped arrows: spermatocytes; solid arrows: spermatids; asterisks: Leydig cells. For each group, the left inset represents SYCP3, the middle inset shows PNA lectin and the right one represents StAR. Scale bars represent 20 μm and 10 μm in the insets. (**C**) Histogram showing the quantification of TFAM fluorescence signal intensity. All values are expressed as the mean ± SD of 5 animals in each group. D-Asp vs. Control (C): * *p* < 0.05.

The expression levels of NRF1 and TFAM, two key markers of mitochondrial biogenesis, were higher in the D-Asp group than in the control group (*p* < 0.05), as shown in Figure 5A. Immunofluorescence staining was conducted to examine the localization of TFAM in the testes of control and D-Asp-treated rats. In the control testes, a TFAM signal was distributed in the cytoplasm of germ cells, specifically, in spermatocytes (striped arrows; inset) and round spermatids (solid arrows); positive staining was also found in the interstitial Leydig cells (asterisks) (Figure 5B). Interestingly, D-Asp induced an increased fluorescent signal, particularly in spermatocytes and Leydig cells as compared to the controls (*p* < 0.05; Figure 5C). In addition, each above-mentioned cell-type was identified in a co-localization analysis of TFAM together with Synaptonemal Complex protein 3 (SYCP3, spermatocytes marker; Figure 5B left small panel); Peanut Agglutinin lectin (PNA), that highlights the acrosome system (spermatids marker; Figure 5B, central small panel); and Steroidogenic Acute Regulatory Protein (StAR, Leydig cells marker; Figure 5B, right small panel).

#### 3.4.3. Effects of D-Asp on Mitochondrial Mass

**Figure 6:**
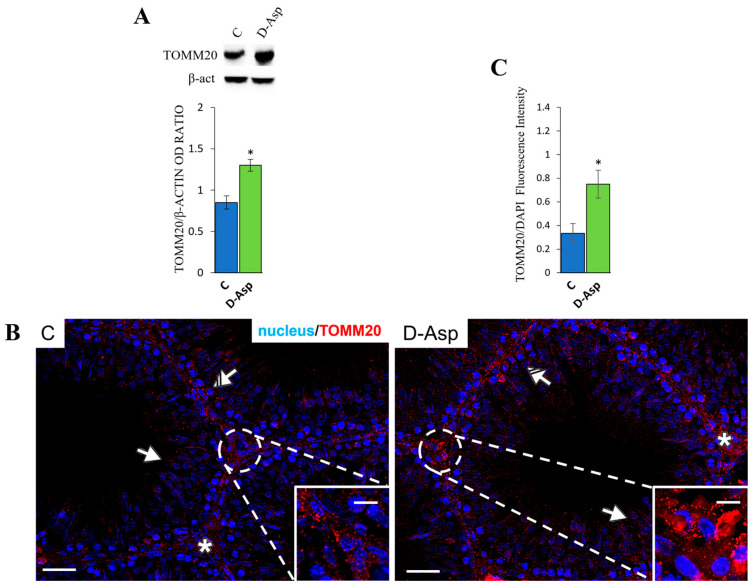
Mitochondrial mass analysis of control and D-Asp-treated rats. (**A**) Western blot analysis of TOMM20 (16 kDa) protein expression levels in the testes of rats treated with D-Aspartate (D-Asp) for 15 days, compared to controls. The expression levels of TOMM20 were quantified using ImageJ and normalized to β-actin (42 kDa). (**B**) Immunofluorescence analysis of TOMM20 (red), and nucleus (blue) of control and D-Asp-treated rats. Striped Arrows: spermatocytes; solid arrows: spermatids; asterisks: Leydig cells. Scale bars represent 20 μm and 10 μm in the insets. Histogram showing the quantification of TOMM20 (**C**) fluorescence signal intensity. All values are expressed as the mean ± SD of 5 animals in each group. D-Asp vs. Control (C): * *p* < 0.05.

The expression levels of TOMM20, a marker of mitochondrial mass, were higher in the D-Asp group than in the control group (*p* < 0.05; Figure 6A), consistent with previous findings regarding the effects of D-Asp on mitochondrial dynamics, which showed that D-Asp favored fusion events (Figure 5). To investigate the localization of TOMM20 in the testes of control and D-Asp-treated rats, an IF staining was performed. As shown in Figure 6B, the TOMM20 signal was diffusely localized in the cytoplasm of control germ cells, specifically in spermatocytes (striped arrows; inset) and round spermatids (solid arrows); a positive staining was particularly found in the interstitial Leydig cells (asterisks). Interestingly, in the testis sections of D-Asp-treated rats, a significant increase in staining was observed in most germ cells, and a strong signal was detected in the cytoplasm of Leydig cells. The analysis of fluorescence intensity confirmed an increase in TOMM20 protein levels (*p* < 0.05) (Figure 6C) in the D-Asp group compared to the control.

## 4. Discussion

Mitochondria and ER interactions constitute a crucial aspect of cellular functionality and regulation. This physical association between the two organelles occurs through MAMs, which are specialized regions that connect the ER and mitochondria. They play a key role in various cellular processes, such as lipid metabolism, calcium signaling, regulation of ER stress, and mitochondrial dynamics. MAMs are also essential for the efficient production of steroid hormones, which regulate reproductive functions. Based on these assumptions, this study aims to evaluate the role of D-Asp in the steroidogenesis of rat testes, focusing on the expression levels of proteins involved in MAM communication and functioning and on mitochondrial dynamics.

High levels of free D-Asp have been detected in the male gonads of vertebrates, and over the last decades, our research group and others have demonstrated that D-Asp plays a role in animal reproduction [13,14,15]. In vivo studies have shown that the administration of D-Asp to rats induces testosterone synthesis and upregulates androgen receptor expression via the NMDA receptor-ERK pathway [19]. Oral treatment with D-Asp has been shown to improve sperm quality in mice [21]. This improvement is due to the active role of D-Asp in spermatogenesis, as demonstrated by the increased expression of two proteins involved in cytoskeleton remodeling, prolyl endopeptidase (PREP) and Disheveled-Associated-Activator of Morphogenesis1 (DAAM1), and essential for gamete differentiation [22,23]. Accordingly, in the present study, we demonstrated that D-Asp administration induced a significant increase in plasma testosterone levels.

In vitro studies have indicated that D-Asp can directly regulate the production of androstenedione and T in both immature [24] and mature Leydig cells [25,26] through the increasing expression of StAR, P450scc (cytochrome P450 cholesterol-side chain cleavage enzyme), and 3β-HSD. Additionally, D-Asp can promote reproduction by directly activating spermatogonial mitosis [13,27,28]. It has also been shown to improve sperm quality and increase in vitro fertilization rates in mice [29]. Further results revealed that D-Asp promotes the proliferation and meiosis of spermatocytes in GC-2 cells through the AMPAR/ERK/Akt pathway. This resulted in increased expression levels of PCNA, p-H3, and SYCP3 proteins, indicating the active involvement of this amino acid in germ cell maturation [30]. Based on these assumptions, our present study aimed first to evaluate the effects of D-Asp treatment on redox balance. Cellular oxidative stress refers to the imbalance between the reactive oxygen species (ROS) production and the cell’s ability to detoxify them. Superoxide dismutase and catalase are two essential enzymes involved in the cellular defense against oxidative stress. The regulation and activity of these enzymes are tightly controlled within cells to maintain redox balance and prevent oxidative stress and damage to proteins, lipids, and DNA. Our results have indicated that in vivo administration of D-Asp significantly stimulated both the enzymatic activity and protein expression levels of the antioxidant enzymes, SOD and CAT. At the same time, it induced a decrease in testicular oxidative status, as evidenced by a significant reduction in TBARS levels.

Cholesterol, the precursor of all steroid hormones, is synthesized in the ER and then distributed to various membranes, including mitochondria, where it is transformed into active steroid hormones. StAR transport protein regulates cholesterol transfer within the mitochondria from the OMM to the IMM. At the inner mitochondrial membrane, the cytochrome P450 cholesterol-side chain cleavage enzyme catalyzes the conversion of cholesterol to pregnenolone. Subsequently, steroid synthesis can proceed through various pathways to produce all steroid hormones. However, several studies have demonstrated that cholesterol is transferred from the ER to mitochondria through the ER–mitochondria contact sites of MAMs. This process involves the protein ATAD3A [31,32], as well as two other proteins, SOAT1/ACAT1 and FACL4, which mediate the lipid transfer. The results of our study showed that the expression levels of all these proteins significantly increased in the testes of rats treated with D-Asp. A confirmation of this result is provided by the immunofluorescence analysis of ATAD3A where the immunopositivity of Leydig cells indicated an increased expression of ATAD3A in the testes of D-Asp-treated rats.

ER is widely recognized as the primary intracellular calcium storage site. However, calcium is also transferred to mitochondria through ER–mitochondria contact sites, where it plays a crucial role in regulating mitochondrial metabolism. On MAMs there are specific channels mediating the uptake of calcium by mitochondria from the ER. Specifically, VDAC forms a complex with the cytosolic chaperone GRP75 that links the ER to mitochondria facilitating calcium mitochondrial uptake [33]. Both cytosolic and mitochondrial calcium levels regulate the early steps of steroidogenesis [34]. Calcium levels stimulate both intramitochondrial cholesterol transfer and the expression of StAR protein. In D-Asp-treated rat testes, we observed a significant increase in both VDAC and GRP75 expression levels. These results suggest that D-Asp increased the expression of these proteins, which promoted calcium influx into the mitochondria, resulting in an improvement in steroidogenesis.

ER stress is determined by the accumulation of not-folded or misfolded proteins, leading to the activation of an unfolded protein response (UPR) [35]. GRP78 is a heat shock protein chaperone and is the master of UPR that facilitates protein folding and prevents the formation of protein aggregates in the ER [7]. In the absence of GRP78, StAR is not correctly folded and is therefore proteolyzed [36], therefore GRP78 playing a crucial role in StAR functioning is consequently also crucial in steroidogenesis. However, the expression of GRP78 is induced in conjunction with ER stress and, therefore, its increased expression is due to this stressful condition. Our results indicated that D-Asp did not induce an ER stress condition, as evidenced by the decreased GRP78 protein expression levels in the rat testes treated with D-Asp.

Mitochondrial dynamics refer to the processes of fusion and fission within cells. The dynamic nature of mitochondria is essential for maintaining their health, function, and distribution within the cell, allowing mitochondrial replication and the repair of defective mitochondria. This mitochondrial plasticity is important in many cellular functions, including hormonal regulation. The regulation of mitochondrial fusion/fission dynamics involves two dynamin-like GTPases, Mitofusin 1 and Mitofusin 2, which modulate interactions between mitochondria and between mitochondria and ER. MFN1 and MFN2 mediate mitochondrial fusion in concert with another GTPase, OPA1, in the IMM. They are widely expressed in several tissues, i.e., brain, liver, adrenal glands, and testes [37]. Mitochondrial fission requires DRP1, a cytoplasmic GTPase that determines the scission of the mitochondrion. Our results showed a significant increase in the protein expression levels of MFN1, MFN2, and OPA1 in the rat testes treated with D-Asp, indicating upregulation of fusion. This result was confirmed by immunofluorescence analysis for MFN1 and MFN2, revealing their localization in Leydig cells. At the same time, a significant decrease in DRP1 expression levels showed that the fission event was reduced in D-Asp rat testes compared to the controls.

Mitochondrial biogenesis is the genesis of new mitochondria within a cell. It involves the synthesis and incorporation of mitochondrial components, such as proteins, lipids, and mtDNA, to create fully functional mitochondria. Mitochondrial biogenesis is crucial for maintaining cellular energy production, adapting to changes in energy demand, and replacing damaged or dysfunctional mitochondria. Mitochondrial genome replication depends on nuclear-gene products and both nuclear and mitochondrial genomes are implicated in the biosynthesis of mitochondria. NRF-1 and TFAM stimulate the expression of nuclear and mitochondrial genes involved in mitochondrial replication. TFAM is a key protein that binds to mtDNA and is essential for the replication and maintenance of the mitochondrial genome. Consistent with the above-reported results on fusion dynamics, the testes of rats treated with D-Asp showed significantly increased expression levels of both NRF1 and TFAM proteins compared to the controls, resulting in biogenesis activation.

Finally, both mitochondrial fusion and biogenesis led to an increased mitochondrial mass, as demonstrated by the increased expression of TOMM20 in rat testes after D-Asp treatment. TOMM20 is a peripheral component of the TOM complex, which acts as a primary receptor for mitochondrial precursor proteins [12]. In our results, the protein expression levels of TOMM20 were significantly higher in the testes of D-Asp-treated rats compared to those in the control groups. Immunofluorescence analyses confirmed the increase in immunopositivity in the Leydig cells of D-Asp-treated rat testes compared to the controls, confirming the increasing mitochondrial mass in these steroidogenic cells by D-Asp.

## 5. Conclusions

In conclusion, this study demonstrated that D-Asp contributes to the functional enhancement of MAM by increasing lipid transfer and calcium signaling from the ER to the mitochondria as well as to the reduction in ER stress, activities of crucial importance for efficient steroidogenesis. Furthermore, the effects of D-Asp on mitochondrial dynamics, i.e., activation of fusion and biogenesis and increase in mitochondrial mass, further support the active role of the amino acid in testicular activity. However, much effort is still needed to understand the mechanisms underlying the contribution of the D-Asp to steroidogenesis/spermatogenesis processes and the potential value of this molecule in overall male fertility.

## Figures and Tables

**Table 1:** List of all the used antibodies.

Antibody	Molecular Weight (kDa)	WB Dilution	IF Dilution	Source
SOD2	22	1:4000	-	Abclonal, Woburn, MA, USA #A1340
CAT	60	1:1000	-	Sigma-Aldrich, St. Louis, MO, USA #C0979
ATAD3A	70	1:1000	1:50	Sigma-Aldrich, St. Louis, MO, USA #SAB3500668
SOAT1/ACAT1	47	1:1000	-	Abcam, Cambridge, UK #ab39327
FACL4	79	1:1000	-	Abcam, Cambridge, UK #ab227256
VDAC	32	1:1000	-	Cell Signaling Technology, Danvers, MA, USA #4661
GRP75	75	1:1000	-	Cell Signaling Technology, Danvers, MA, USA #2816
GRP78	78	1:1000	-	Cell Signaling Technology, Danvers, MA, USA #3183
MFN1	84	1:1000	1:100	Abcam, Cambridge, UK #ab221661
MFN2	86	1:500	1:50	Abcam, Cambridge, UK #ab124773
OPA1	85-112	1:1000	-	Abcam, Cambridge, UK #ab42364
DRP1	78-82	1:1000	-	Cell Signaling Technology, Danvers, MA, USA #8570
NRF1	56	1:1000		Cell Signaling Technology, Danvers, MA, USA #69432
TFAM	24	1:1000	1:100	Abcam, Cambridge, UK #ab131607
SYCP3	30-33	-	1:100	Santa Cruz Biotechnology, Santa Cruz, CA, USA #sc74569
PNA Lectin Alexa Fluor 568	-	-	1:50	Thermo Fisher Scientific, Waltham, MA, USA #L32458
StAR	32	-	1:100	Santa Cruz Biotechnology, Santa Cruz, CA, USA #sc-166821
TOMM20	16	1:500	1:100	Sigma-Aldrich, Milan, Italy WH0009804M1
β-Actin	42	1:2000	1:100	Elabscience Biotechnology, Wuhan, China #E-AB-20031
Goat anti-Rabbit IgG HRP	-	1:3000	-	Vector Laboratories, Burlingame, CA, USA #PI-1000
Goat anti-Mouse IgG HRP	-	1:2000	-	BioActs, Namdong-gu, Incheon, Korea #RSA1122
Goat anti-Rabbit Alexa Fluor 488	-	-	1:500	Thermo Fisher Scientific, Waltham, MA, USA #A32731
Goat anti-Mouse CF^TM^ 568	-	-	1:500	Sigma-Aldrich, Saint Louis, MO, USA #SAB4600082

## Data Availability

The data presented in this study are available on reasonable request from the corresponding author.
